# Relationships Between Alexithymia and Psychopathy in Heroin Dependent Individuals

**DOI:** 10.3389/fpsyg.2019.02269

**Published:** 2019-10-09

**Authors:** Elena Psederska, Svetoslav Savov, Nikola Atanassov, Jasmin Vassileva

**Affiliations:** ^1^Department of Cognitive Science and Psychology, New Bulgarian University, Sofia, Bulgaria; ^2^Bulgarian Addictions Institute, Sofia, Bulgaria; ^3^Department of Psychiatry, Virginia Commonwealth University, Richmond, VA, United States; ^4^Institute for Drug and Alcohol Studies, Virginia Commonwealth University, Richmond, VA, United States

**Keywords:** alexithymia, psychopathy, substance use disorders, opioid use disorder, emotion processing

## Abstract

**Background:**

Psychopathy and substance use disorders are highly co-morbid and their co-occurrence is associated with higher severity of addictive behavior and increased risk of violent offending. Both substance use disorders and psychopathy are related to prominent impairments in emotion processing, which are also central features of alexithymia. The nature of the relationship between psychopathy and alexithymia is not well-understood and has been particularly understudied among substance dependent individuals.

**Aim:**

Our goal was to evaluate the levels of psychopathy and alexithymia in a relatively homogeneous sample of heroin dependent individuals (HDIs) and healthy controls and to examine group differences in the pattern of associations between these constructs.

**Methods:**

We examined 62 participants (31 heroin dependent individuals and 31 healthy controls) with the Psychopathy Checklist: Screening version (PCL:SV, Hart et al., 1995) and the Toronto Alexithymia Scale-20 (TAS-20, Bagby et al., 1994).

**Results:**

Heroin dependent individuals were characterized by higher levels of both psychopathy and alexithymia as compared to the control group. In addition, HDIs with higher levels of psychopathy reported more difficulties in identifying and verbalizing emotional states. In the heroin group, alexithymia was more strongly associated with the impulsive/antisocial characteristics (impulsivity, irresponsibility, antisocial behavior) than with the interpersonal/affective features of psychopathy (grandiosity, manipulativeness, lack of empathy, and remorse).

**Conclusion:**

Our findings suggest that alexithymia may be one potential mechanism linking psychopathy with opioid use disorders. The development of interventions targeting alexithymia could have significant applications in relapse prevention programs and psychotherapy of substance use disorders with concurrent psychopathy.

## Introduction

### Substance Use Disorders

Substance use disorders (SUDs) are among the most prevalent mental health problems worldwide. Chronic and excessive drug use results in severe impairments in key aspects of individual’s functioning, with most prominent disturbances noted in the social, legal, medical and psychological domains (United Nations Office on Drugs and Crime, 2017). SUDs are among the most challenging psychiatric disorders, associated with frequent treatment drop-outs (Brorson et al., 2013) and increased risk of post-treatment relapse (Andersson et al., 2019), which are particularly alarming in opioid use disorder (Smyth et al., 2010). In addition, SUDs often co-occur with other mental disorders, which further exacerbates the course and prognosis of treatment (Buckley, 2006). Recently, research has focused increasingly on the heterogeneity of SUDs with regards to different stages of the addiction cycle, substance-specific vulnerability factors, and premorbid personality risk factors. Understanding the heterogeneity of addictions is pivotal, as it may have important implications for prevention and treatment of SUDs and lead to the development of more effective interventions based on individual differences in addictive behavior.

Contemporary neurobiological models conceptualize addiction as a chronic relapsing disorder, characterized by a compulsion to seek and use the drug, loss of control over drug use, and emergence of negative emotional states (Koob and Volkow, 2010). Addiction is proposed to evolve along three stages: *binge/intoxication, preoccupation/anticipation (craving)*, and *withdrawal/negative affect* (Koob and Volkow, 2010). The transition between these stages is associated with a shift from positive reinforcement mechanisms in the earlier stages of addiction, related to appetitive motivation and dopaminergically-mediated reward, to negative reinforcement “antireward” mechanisms (Koob and LeMoal, 2008) in the later stages of addiction, mediated by brain stress systems (Koob, 2008). These latter stages are associated with negative emotional states such as dysphoria, depression, anxiety, anhedonia, and alexithymia, defined as the “dark side” of addiction (Koob and Volkow, 2010; Kwako et al., 2016). Every stage of the addiction cycle is mediated by different neurocircuits and is associated with distinct brain regions involved in the transition between stages (Koob and Volkow, 2010).

Early psychobiological theories viewed addiction as a unitary phenomenon. In contrast, recent models of addiction and empirical findings from the neurocognitive, neuroimaging, and genetic literatures emphasize fundamental differences between different types of SUDs, explained by the unique effects of specific classes of drugs (for review see, Badiani et al., 2011). Accordingly, Wise and Koob (2014) have proposed that the three main stages of addiction are differentially related to different classes of drugs. Specifically, psychostimulant addiction is strongly related to the binge/intoxication stage where positive reinforcement mechanisms predominate. In contrast, opioid addiction is associated more strongly with the withdrawal/abstinence stage, characterized by negative reinforcement mechanisms (Wise and Koob, 2014). Excessive use of opioids in particular is proposed to be uniquely related to severe impairments in the homeostatic regulation of emotional behavior leading to neuroadaptive processes in brain reward systems, characterized by chronic hypersensitivity to painful emotional states (hyperkatifeia) (Shurman et al., 2010).

In addition to the heterogeneity of addiction based on specific drug class, current models in the field emphasize the importance of different addiction profiles based on key personality risk factors (Gudonis et al., 2009; Castellanos-Ryan and Conrod, 2012). Modern personality-based approaches to SUDs have suggested that specific externalizing (impulsivity-related) and internalizing (related to negative affect) personality traits are among the most salient vulnerability factors for drug addiction (Gudonis et al., 2009; Castellanos-Ryan and Conrod, 2012). These personality dimensions have been differentially associated with vulnerability to different types of SUDs. For example, psychostimulant dependence was uniquely associated with externalizing traits such as impulsivity, whereas opioid dependence was more strongly related to internalizing traits such as anxiety sensitivity and hopelessness (Conrod et al., 2000; Woicik et al., 2009). Moreover, research suggests that the impact of personality factors is not limited to the etiology of SUDs, but also plays a key role in the maintenance of addictive behavior and has a negative impact on treatment outcomes for substance dependent individuals (SDIs) (for review see Staiger et al., 2007). For example, personality traits such as impulsivity and sensation seeking have been linked to elevated treatment drop-out rates (Patkar et al., 2004), whereas negative affectivity has been associated with increased risk of relapse (Witkiewitz and Villarroel, 2009). Thus, identification of specific pretreatment personality characteristics that increase the risk of relapse is important for the development of personality matched interventions and could lead to a significant improvement in the treatment outcomes for SDIs, facilitating treatment motivation and reducing relapse rates.

### Psychopathy and Substance Use Disorders

Psychopathy is a personality construct with increasing importance in the etiology and prognosis of SUDs. Psychopathy is defined as an extreme variant of antisocial personality disorder, consisting of a constellation of affective, interpersonal and behavioral characteristics (Hare and Neumann, 2006). Early theories of psychopathy distinguish between primary (callous/unemotional) and secondary (antisocial/impulsive) subtypes of psychopathic individuals (Karpman, 1948; Porter, 1996). Both psychopathy subtypes share common traits and behaviors such as hostility, aggression, and antisocial behaviors. However, primary psychopaths are characterized by grandiosity, superficiality, lack of empathy and remorse, and low to moderate levels of anxiety, whereas secondary psychopaths are more anxious, with high levels of negative affectivity and emotional disturbances (Blackburn and Coid, 1998; Vassileva et al., 2005; Poythress and Skeem, 2006). This distinction is supported by studies with the Psychopathy Checklist-Revised (PCL-R; Hare, 1991, 2003), the most widely used instrument for measuring psychopathy. Psychometric analyses of the PCL-R identify a two-factor solution, which differentiates between interpersonal/affective (manipulative, lack of remorse, lack of empathy) and impulsive/antisocial features of psychopathy (impulsive, irresponsible, antisocial), closely resembling the traditional distinction between primary and secondary psychopathy (Harpur et al., 1989; Hare et al., 1990). In addition, studies with the PCL-R identify distinctive patterns of relationships between the two PCL-R factors and various psychological constructs. Factor 1, measuring the interpersonal and affective features of psychopathy, has been uniquely related to attenuated levels of both anxiety and impulsivity, whereas Factor 2, reflecting the impulsive and antisocial lifestyle dimension of psychopathy, has been associated with negative emotionality, impulsivity, anger, and substance use (Hare, 1991; Hicks et al., 2004; Snowden and Gray, 2011).

Substance use disorders are among the most prevalent disorders comorbid with psychopathy (Smith and Newman, 1990; Crocker et al., 2005). The rate of SUDs has consistently been found to be higher among psychopaths than among non-psychopaths in criminal samples (Smith and Newman, 1990; Blackburn and Coid, 1998; Rasmussen et al., 1999). Similarly, the rates of psychopathy in SDIs are significantly higher than those found in the general population (Rutherford et al., 2000). Moreover, psychopathy has also been shown to exacerbate some neurocognitive deficits of SDIs, particularly those related to impulsive decision-making (Vassileva et al., 2007, 2011) and has also been associated with increased lifetime sexual HIV risk behaviors among SDIs (Wilson and Vassileva, 2016). Studies implementing machine-learning techniques have found that psychopathy was the only common predictor of substance dependence on different classes of drugs (heroin, amphetamine, cannabis, nicotine, and alcohol) (Ahn and Vassileva, 2016; Vassileva et al., 2019). These findings suggest that psychopathy may be among the key diagnostic markers for drug addiction, which is reflected by the recent inclusion of psychopathy measure in the phenotypic battery recently proposed by the National Institute on Drug Abuse (NIDA) (Ramey, 2017).

Regarding the heterogeneity of psychopathy, substance use has been more strongly associated with the impulsive/antisocial features of psychopathy and weakly or non-related to the core interpersonal/affective dimension of the disorder (Hart and Hare, 1989; Smith and Newman, 1990; Hemphill et al., 1994). In the context of the heterogeneity of addiction, studies have found that heroin dependent individuals (HDIs) are characterized by higher levels of psychopathy than amphetamine dependent individuals (Ahn et al., 2014; Bozgunov et al., 2014; Ahn and Vassileva, 2016). These findings could be explained by the severe social disadaptation and higher rates of criminal offending observed in opioid addiction relative to other types of SUDs (Bennett et al., 2008; Bennett and Holloway, 2009).

The high comorbidity between psychopathy and SUDs has significant implications for the course and treatment outcomes of SUD. Research shows that problematic drug use is much more difficult to treat in SDIs with high levels of psychopathy, which are associated with more relapses and increased risk for violent offending (Smith and Newman, 1990; Alterman et al., 1998). Psychopathy has been consistently associated with negative treatment outcomes in SDIs, including high attrition rates, substance use during treatment, high relapse rates and post-treatment offending (O’Neill et al., 2003; Richards et al., 2003). These studies have led to a rather pessimistic stance among professionals regarding treatment efficacy for SDIs with concurrent psychopathy, who have been portrayed as treatment-resistant (Gudonis et al., 2009). However, some authors suggest that the identification of specific underlying common factors related to the parallel development of psychopathy and SUDs could facilitate treatment planning by informing personality-matched treatment strategies, which could in turn improve treatment efficacy (Gudonis et al., 2009). The identification of common vulnerability factors should address both the heterogeneity of SUDs related to different stages of the addiction cycle and the heterogeneity related to use of specific classes of drugs, as well as the distinction between interpersonal/affective and impulsive/antisocial features of psychopathy. Disinhibition and negative affectivity are among the most influential candidate personality traits, proposed to affect both comorbidity rates and treatment efficacy of concurrent psychopathy and SUDs. Although personality traits related to the broader dimension of disinhibition such as impulsivity, sensation seeking, and novelty seeking have long been linked to the parallel development of psychopathy and SUDs (Krueger et al., 2002; Patrick et al., 2005), recent studies have focused increasingly on the importance of negative affectivity and related emotional deficits in explaining the co-occurrence of the two disorders (Gudonis et al., 2009). This shift of focus was influenced by recent studies that conceptualize impulsivity as a result of emotion dysregulation and suggesting that impulsive behaviors are largely driven by difficulties in regulating negative affect (Cooper et al., 2000; Weiss et al., 2012). In addition, the role of emotion dysregulation has been increasingly emphasized in the understanding psychopathy (Garofalo et al., 2018). Therefore, factors related to the broader dimension of emotion regulation could be of particular importance for understanding the comorbidity between psychopathy and SUDs.

### Deficits in Emotion Regulation as a Common Factor Linking Psychopathy and SUDs

Both psychopathy and SUDs have been linked to prominent deficits in emotion processing (Kornreich et al., 2003; Kirsch and Becker, 2007). Early theories of psychopathy have proposed that lack of emotion was one of the central features of the disorder (Cleckley, 1976). Initial studies have portrayed psychopathic individuals as fearless and devoid of normal emotional experience (Lykken, 1957; Patrick, 1994; Blair, 1995) and this was especially true for individuals high on primary psychopathy. Although the interpersonal/affective features of psychopathy (reflecting primary psychopathy) have been uniquely related to deficient emotion processing as measured on laboratory tasks (Dawel et al., 2012), some findings suggest that psychopathic individuals are skillful at masking their affective deficits by identifying and simulating wide range of emotions in order to manipulate others (Del Gaizo and Falkenbach, 2008). However, recent studies have challenged this traditional view by suggesting that psychopathic individuals experience emotions, but have difficulties regulating them (Garofalo et al., 2018). This notion was supported by studies reporting that difficulties in emotion regulation were not limited to the impulsive/antisocial features of psychopathy but also have a key role in predicting the affective traits associated with psychopathy (Garofalo et al., 2018). In addition, accumulating data from experimental studies suggest that psychopathic individuals can show normal emotional experience under specific contexts (Newman et al., 2010; Drayton et al., 2018). These latter studies emphasize the importance of attention and motivation in performing emotional tasks and suggest that psychopaths are capable of experiencing and processing different emotions when this is in service of their current goals (Groat and Shane, 2019).

Problem substance use has long been linked to emotion dysregulation (Kober and Bolling, 2014). One of the most widely accepted theories regarding the etiology of drug addiction suggests that it is the result of severe deficits in emotion regulation abilities where the substance represents an alternative, external strategy for regulating unbearable affects (Cooper et al., 1995; Khantzian, 2003). Studies have consistently found that SUDs are related to a wide range of difficulties in experiencing and processing emotions (Kornreich et al., 2003; Hoshi et al., 2004; Verdejo-García et al., 2007) and to disrupted emotion regulation abilities (Ghalehban and Besharat, 2011; Dingle et al., 2018).

Since emotion dysregulation appears to represent a common vulnerability factor for both psychopathy and SUDs, difficulties in emotion regulation could be considered a potential common underlying mechanism explaining the co-occurrence between the two disorders. In this context, the identification of more narrowly defined risk factors related to difficulties in emotion processing and regulation could be of particular importance when studying the relationship between psychopathy and SUDs.

### Alexithymia as Potential Common Mechanism Linking Psychopathy and SUDs

Alexithymia is a narrower construct, which has been consistently associated with poor emotion regulation abilities and difficulties in emotion processing. Alexithymia reflects a constellation of deficits in emotion processing and self-regulation, such as limited emotional repertoire, inability to differentiate nuances of feelings, and difficulties in describing and interpreting emotions (Sifneos, 1973).

Studies have consistently found that SDIs are characterized by higher levels of alexithymia (Pinard et al., 1996; Cleland et al., 2005). The prevalence of alexithymia in SDIs is almost twice as high as compared to healthy controls (Farges et al., 2004). Empirical studies show that levels of alexithymia in SDIs do not decrease significantly even after long periods of abstinence, supporting the idea that alexithymia is a stable personality characteristic of addictive behavior (Pinard et al., 1996; Morie et al., 2015), which may be especially pronounced in the latter stages of addiction, such as withdrawal and protracted abstinence (Koob and Volkow, 2010). Most of the studies investigating the relationship between alexithymia and drug addiction have focused on individuals with alcohol or polysubstance dependence (Haviland et al., 1994; Cleland et al., 2005; Ghalehban and Besharat, 2011; Morie et al., 2016; Cruise and Becerra, 2017); therefore, our understanding of the association of alexithymia with different classes of drugs remains limited. Given that opioid addiction is strongly associated with negative reinforcement mechanisms and sensitivity to negative emotional states, alexithymia may play more central role in opioid addiction and the withdrawal/abstinence stage characterized by negative emotionality.

Recent studies of psychopathy have focused increasingly on the role of alexithymia and have reported pronounced difficulties in identifying emotions among violent offenders (Garofalo et al., 2017; Gillespie et al., 2018; Mayer et al., 2018). Thus, alexithymia appears to represent a common risk factor for both psychopathy and SUDs and can be considered a potential common underlying mechanism leading to their frequent co-occurrence. With regards to the heterogeneity of psychopathy, Lander et al. (2012) suggest that alexithymia would be uniquely associated with the impulsive/antisocial features of psychopathy, related to higher levels of negative affectivity, emotional lability and emotional difficulties (Hicks et al., 2004; Vidal et al., 2010; Kimonis et al., 2012). In contrast, they expect that alexithymia would be negatively associated or non-related to the interpersonal/affective traits, proposed to reflect the core affective deficit of psychopathy. Results to date have been contradictory and inconsistent, with some studies indicating positive relationships between alexithymia and the impulsive/antisocial traits (Kroner and Forth, 1995; Louth et al., 1998) but negative (Kroner and Forth, 1995) or no relationship (Louth et al., 1998) with the interpersonal/affective features of psychopathy. Other studies fail to find any relationship between alexithymia and psychopathy (Möller et al., 2014) or identify fully positive (Grieve and Mahar, 2010) or fully negative (Pham et al., 2010) correlations between the two constructs. The nature of the relationship between psychopathy and alexithymia is not well-understood and has been particularly understudied among SDIs. To our knowledge, only one study to date has addressed the relationship between psychopathy and alexithymia in SDIs, which found positive correlation between the two constructs (Gori et al., 2014). However, participants in Gori et al. (2014) study consisted of individuals diagnosed with different types of SUDs (e.g., alcohol-, amphetamine-, heroin use disorder, etc.), which prevents the identification of possible substance-specific associations between the two constructs. Since opioid addiction is associated both with higher levels of psychopathy and predominance of negative reinforcement mechanisms, our goal was to examine the relationship between alexithymia and psychopathy among HDIs. If alexithymia proves to be an important underlying mechanism linking psychopathy and opioid addiction, treatments targeting alexithymia may be successful in reducing relapse rates and post-treatment offending that are common in individuals with opioid use disorders (Alterman et al., 1998).

The aims of the present study were to investigate levels of alexithymia and psychopathy and their associations among HDIs and healthy controls. Our main hypothesis was that psychopathy and alexithymia would be positively associated in HDIs. Specifically, we hypothesize that alexithymia would be more strongly associated with the impulsive features of psychopathy (impulsivity, irresponsibility, antisocial behavior, reduced inhibitory control), reflecting secondary psychopathy than with the interpersonal/affective features of psychopathy (grandiosity, manipulative behavior, inability to experience guilt, and empathy), reflecting primary psychopathy.

## Materials and Methods

### Participants and Data Collection

Participants were recruited as part of a larger ongoing study in Bulgaria investigating different types of impulsivity in substance dependent individuals. The testing was conducted by an experienced team of psychologists at the Bulgarian Addictions Institute in Sofia, Bulgaria. The study was approved by the Institutional Review Boards of Virginia Commonwealth University and the Medical University in Sofia on behalf of the Bulgarian Addictions Institute.

Participants were recruited from substance abuse clinics and therapeutic communities in Bulgaria as well as through the study’s web page and Facebook page. All participants had to meet the following inclusion criteria: minimum of 8^th^ grade education, being fluent in Bulgarian, IQ higher than 75, no history of neurological or psychotic disorder, no history of traumatic brain injury. All participants were abstinent at the time of testing, as verified by urine toxicology screen and alcohol breathalyzer test. All participants signed a consent form before taking part in the study. The current sample consisted of 62 participants (42 males, 20 females) of whom 31 (21 males, 10 females) met DSM-IV criteria for lifetime mono-dependence on heroin and 31 (21 males, 10 females) were healthy controls with no history of substance abuse or dependence. All participants were between the ages of 20 and 45. Participants in the two groups were well-matched on demographic characteristics, except on years of education, where HDIs had significantly fewer years of education than controls [*t*(60) = 4.688, *p* = 0.000]. Descriptive statistics and group differences on demographic variables are presented in Table 1.

**TABLE 1:** Descriptive statistics and group differences in demographic variables.

	**Control group**	**Heroin dependent group**	***p***
*N*	31	31	
Age	29.97 (5.78)	31.58 (4.92)	0.241
Years Education	**15.39 (2.40)**	12.55 (2.36)	**0**.**000**
Estimated IQ	109.74 (14.17)	105.55 (12.52)	0.222

*Results are presented as means (SD). Values in bold are significant.*

All participants in the heroin dependent group were in protracted abstinence at the time of testing: 26 were in full sustained remission for more than 1 year and 5 were in early full remission for less than 1 year. The average duration of remission was 5.79 (SD = ± 5.84) years. The average duration of opiate use was 6.76 years (SD = ± 3.19). On average, participants met 6.06 (SD = ± 1.15) criteria for lifetime opiate dependence. The group of the SDIs in the current sample consisted of individuals with lifetime mono-dependence on heroin who met criteria for heroin dependence but had no history of dependence on other drugs.

### Measures and Procedures

Measures were administered as part of a larger assessment battery administered over two testing sessions with approximate duration of 3 h each, conducted on two separate days. The assessment battery consisted of clinical interviews, self-report questionnaires and computer-based neurocognitive tests. The first session included assessment of DSM-IV substance abuse and dependence, externalizing psychopathology (e.g., psychopathy, ASPD, ADHD) and IQ estimation. The second session included neurocognitive tasks, and self-report measures of personality and internalizing psychopathology (anxiety, depression, anxiety sensitivity, alexithymia).

#### Structured Clinical Interview for DSM-IV – Substance Abuse Module (SCID-SAM; First et al., 1996)

The SCID is a semi-structured clinical interview designed to determine whether an individual meets criteria for any SUD (alcohol-, cannabis-, stimulant-, hallucinogen-, opioid use disorders) according to the DSM-IV. Raters assess the presence of DSM-IV symptoms of substance abuse and dependence using a three-point scale (0 = not present, 1 = subthreshold, 2 = present). A diagnosis of substance dependence is made if the participant displayed three (or more) of the substance dependence criteria within a 12-month period.

#### Psychopathy Checklist: Screening Version (PCL:SV; Hart et al., 1995)

The PCL:SV is a 12-item semi-structured interview based on the Psychopathy Checklist – Revised (PCL-R, Hare et al., 1990) used for assessment of psychopathy in community and non-forensic samples. The PCL:SV is comprised of two factors assessing characteristics related to primary and secondary psychopathy. Factor 1 consists of six items reflecting the interpersonal and affective characteristics of psychopathy (grandiosity, manipulativeness, lack of empathy, lack of remorse), while the remaining six items from Factor 2 measure an unstable lifestyle and antisocial behaviors (impulsivity, irresponsibility, poor behavioral controls, antisocial behavior in adolescence and adulthood). Items are scored on a three-point scale (0 = absent, 1 = somewhat present, 2 = definitely present) and summed to form a total score ranging from 0 to 24 points. An in-depth psychometric analysis of the Bulgarian version of the PCL:SV with a subset of the current participants was performed by Wilson et al. (2014), which revealed good fit and internal consistency ranging from acceptable to good for the total PCL:SV (α = 0.80), Factor 1 (α = 0.80) and Factor 2 (α = 0.67). Interrater reliability was good for the total PCL:SV (ICC = 0.96), Factor 1 (ICC = 0.93) and Factor 2 (ICC = 0.81). Table 2 displays the internal consistency (Cronbach’s alpha and mean inter-item correlations) of the PCL:SV factors and total score both in the current total sample and separately for HDIs and control participants. Overall, the PCL:SV exhibited acceptable internal consistency with Cronbach’s alphas ranging from 0.72 to 0.90 across groups. The only exception was the PCL:SV Factor 1, which exhibited poor internal consistency in the control group (α = 0.55). In addition to the Cronbach’s alphas, we also examined the mean inter-item correlations, proposed to be a useful measure of internal consistency and scale homogeneity (Clark and Watson, 1995). In our sample, the mean inter-item correlations of the PCL:SV factors and total score fall between 0.20 and 0.39 across groups, which was within the recommended by Clark and Watson (1995) range of 0.15–0.50.

**TABLE 2:** Internal consistency of the PCL:SV and the TAS-20 in the current sample.

**Measure**	**Sample**	**Raw alphas**	**Mean inter-item correlation**
PCL:SV Factor 1	Controls	0.55	0.20
	HDIs	0.79	0.39
	Total	0.79	0.36
PCL:SV Factor 2	Controls	0.75	0.36
	HDIs	0.72	0.30
	Total	0.87	0.53
PCL:SV Total	Controls	0.81	0.28
	HDIs	0.80	0.26
	Total	0.90	0.40
TAS-20 DIDF	Controls	0.85	0.27
	HDIs	0.82	0.33
	Total	0.87	0.35
TAS-20 EOT	Controls	0.34	0.11
	HDIs	0.53	0.20
	Total	0.44	0.15
TAS-20 Total	Controls	0.77	0.16
	HDIs	0.83	0.21
	Total	0.83	0.20

#### Toronto Alexithymia Scale – 20 (TAS-20; Bagby et al., 1994)

The TAS-20 is a self-report measure commonly used for indexing alexithymia. The scale consists of 20 items measured on a five-point Likert scale (from *strongly disagree* to *strongly agree).* The original scale (Bagby et al., 1994) was developed to reflect the three-factor model of alexithymia with 7 items assessing *difficulties in identifying feelings*, 5 items measuring *difficulties in describing feelings* and 8 items reflecting *externally oriented thinking*. Psychometric analysis of the Bulgarian version of the TAS-20 with a subset of the current sample identified a two-factor solution where the first factor reflects *difficulties identifying and describing feelings (DIDF)* and the second factor describes *externally oriented thinking (EOT)* (Popov et al., 2016). The Bulgarian version of the TAS-20 had adequate internal consistency for the total scale (α = 0.86) and Factor 1 *DIDF* (α = 0.89), and acceptable internal consistency for Factor 2 *EOT* (α = 0.69). Table 2 presents the internal consistency (Cronbach’s alpha and mean inter-item correlations) of the TAS-20 factors and total scores both in the current total sample and separately for HDIs and control participants. The internal consistency of the TAS-20 DIDF and the TAS-20 total score ranged from acceptable to good across samples (α = 0.77–0.87, *r_*mean inter–*__*item*_* from 0.16 to 0.35). Consistent with prior research (Bagby et al., 1994; Loas et al., 2001), the reliability of the TAS-20 EOT subscale was poor with α’s ranging from 0.34 to 0.53 and *r*_*mean inter–item*_ from 0.11 to 0.20 across samples.

## Results

All Analyses Were Conducted Using SPSS Version 19.0. Table 3 displays the descriptive statistics of psychopathy and alexithymia for each group. Overall, HDIs scored significantly higher than controls on both psychopathy and alexithymia.

**TABLE 3:** Descriptive statistics and group differences in psychopathy and alexithymia.

	**Control group**	**Heroin dependent group**	***p***
*N*	31	31	
PCL: SV F1	2.06 (1.91)	**5.61 (2.79)**	**0**.**000**
PCL: SV F2	1.61 (2.19)	**7.50 (2.81)**	**0**.**000**
PCL: SV Total	3.68 (3.68)	**13.16 (4.89)**	**0**.**000**
TAS-20 DIDF	27.00 (6.36)	**33.55 (7.83)**	**0**.**001**
TAS-20 EOT	11.19 (2.14)	11.03 (2.42)	0.782
TAS-20 Total	38.19 (7.03)	**44.58 (8.72)**	**0**.**002**

*Results are presented as means (SD). Values in bold are significant.*

The assumption of homogeneity of variance was not met for the psychopathy measure (PCL:SV), therefore a non-parametric analysis (Mann–Whitney) was used to test for group differences. The analysis revealed that the groups differed in all PCL:SV scores: Factor 1, *U* = 145.5, *z* = −4.752, *p* < 0.001; Factor 2, *U* = 48.5, *z* = −6.054, *p* < 0.001; PCL:SV Total, *U* = 63.5, *z* = −5.883, *p* < 0.001, where HDIs scored significantly higher than controls.

The assumption of homogeneity of variance was met for the alexithymia measure (TAS-20), therefore we used Independent Sample *t*-test to test for group differences. HDIs had higher total scores on the TAS-20, *t*(60) = −3.176, *p* < 0.002, as well as on the *difficulties in identifying and describing feelings* (DIDF) subscale, *t*(60) = −3.614, *p* < 0.001. There were no group differences on the TAS-20 *externally oriented thinking* (EOT) subscale, *t*(60) = 0.279, *p* = 0.782.

We then conducted correlational analyses to evaluate the relationship between psychopathy and alexithymia. Zero-order correlations were calculated between PCL:SV scores and TAS-20 scores for each group. In the control group there were no significant correlations between psychopathy and alexithymia. In the heroin group, PCL:SV Factor 1 and PCL:SV total score were positively correlated with the two TAS-20 subscales and the total score. There was also a significant positive correlation between PCL:SV Factor 2 and TAS-20 total score and TAS-20 *difficulties in identifying and describing feelings* subscale, but there was no correlation between PCL:SV Factor 2 and TAS-20 *externally oriented thinking* subscale (Table 4). Additional analyses were conducted to test for differences between the correlations found in the control group and in HDIs (Preacher, 2002). Although the associations between psychopathy and alexithymia were not significant in the control group, the correlation coefficients reflecting the relationship between alexithymia and psychopathy were equal in HDIs and controls (*p* > 0.05).

**TABLE 4:** Zero-order and partial correlations between alexithymia and psychopathy.

	**PCL:SV F1**	**PCL:SV F2**	**PCL:SV Total**
**Control group**			
TAS-20 DIDF	0.194 (0.024)	0.154 (0.188)	0.198
TAS-20 EOT	0.188 (−0.059)	0.381 (0.323)	0.309
TAS-20 Total	0.194 (0.005)	0.216 (0.270)	0.231
**Heroin dependent group**			
TAS-20 DIDF	**0.395^∗^** (0.227)	**0.492^∗∗^ (0.382^∗^)**	**0.523^∗∗^**
TAS-20 EOT	**0.403^∗^** (0.260)	0.269 (0.115)	**0**.**397^∗^**
TAS-20 Total	**0.466^∗∗^** (0.283)	**0.450^∗∗^ (0.383^∗^)**	****0**.**580^∗∗^****

*Results are presented as zero-order correlations (partial correlations). ^∗∗^Correlation is significant at the 0.01 level (two-tailed). ^∗^Correlation is significant at the 0.05 level (two-tailed). Values in bold are significant.*

Partial correlations were calculated to evaluate the unique contributions of each PCL:SV factor in the relationship between psychopathy and alexithymia in HDIs after accounting for the effect of the other factor. After controlling for the effect of PCL: SV Factor 2, the correlations between TAS-20 scores and PCL:SV Factor 1 were no longer significant (*p* > 0.05). In contrast, the correlations between TAS-20 scores and PCL:SV Factor 2 remained significant after controlling for the effect of PCL:SV Factor 1 (Table 4).

## Discussion

The aims of the present study were to compare levels of psychopathy and alexithymia among HDIs and controls, and to examine the pattern of associations between psychopathy and alexithymia. To our knowledge, this is the first study which examined levels of psychopathy, alexithymia and their specific association in ‘pure’ opiate users in protracted abstinence. Thus, our findings are a first step in gathering more knowledge about the unique associations of opiate use (independent of other drugs) and about the protracted abstinence, one of the least well-understood stages of the addiction cycle.

Our results reveal that HDIs exhibit higher levels of psychopathy and alexithymia than healthy controls, even after a prolonged period of abstinence. The group differences in alexithymia were related specifically to difficulties in identifying and verbalizing emotional states but not to externally oriented thinking. Current theories of addiction consider impairments in emotion processing, such as alexithymia, as important underlying risk factors predisposing to development and maintenance of SUDs, which are particularly relevant in the withdrawal/abstinence stage of addiction and are especially pronounced in opioid addiction (Shurman et al., 2010; Wise and Koob, 2014; Kwako et al., 2016). This is supported by our findings, which suggest that alexithymia may be a stable personality feature of HDIs that persists in protracted abstinence.

Another important finding from our study was that psychopathy and alexithymia were positively correlated in HDIs, whereas they were not significantly correlated in non-substance dependent controls. However, surprisingly, the correlation coefficients reflecting the association between psychopathy and alexithymia were not significantly different in HDIs as compared to control participants. The lack of significant association between psychopathy and alexithymia in the control group could be explained, in part, by the relatively little variance in PCL:SV scores in control participants. On the other hand, the group differences in the pattern of associations between psychopathy and alexithymia suggest that their relationship could be influenced by sample-specific characteristics. The majority of studies have examined the link between psychopathy and alexithymia among students and criminal offenders. Studies with offenders have reported inconsistent and mixed findings, with some studies not finding any relationship between alexithymia and psychopathy (Möller et al., 2014) whereas others identifying positive (Kroner and Forth, 1995; Louth et al., 1998; Mayer et al., 2018) or negative (Pham et al., 2010) correlations between the two constructs. These inconsistencies could be due to the heterogeneity in offender samples in these studies with regards to gender [male (Kroner and Forth, 1995; Pham et al., 2010; Möller et al., 2014; Mayer et al., 2018) vs. female (Louth et al., 1998)], type of crimes committed [violent (Kroner and Forth, 1995; Mayer et al., 2018) vs. non-violent, including drug-related crimes (Möller et al., 2014)], and comorbidity with psychiatric disorders (Pham et al., 2010). Studies examining the relationship between psychopathy and alexithymia among students consistently find positive correlations between the two constructs (Grieve and Mahar, 2010; Lander et al., 2012); however, these findings are not representative of the general population (Hanel and Vione, 2016). In addition, the inconsistencies between studies could be due to differences in the way psychopathy was measured. Some studies have used interview-based measures such as the PCL-R or the PCL:SV (Kroner and Forth, 1995; Louth et al., 1998; Pham et al., 2010; Möller et al., 2014), while others have administered self-report measures of psychopathy (Grieve and Mahar, 2010; Lander et al., 2012; Mayer et al., 2018).

To our knowledge, the only study, which explored the correlations between these constructs in a sample of SDIs (Gori et al., 2014) found positive relationships between psychopathy as measured by the Psychopathic Personality Inventory – Revised (PPI-R; Lilienfeld and Widows, 2005) and alexithymia, assessed with the TAS-20. Our results are in line with these findings and suggest that psychopathy and alexithymia play a key role in SUDs. On the basis of the PPI-R total score, Gori et al. (2014) divided their sample into two categories – psychopathic (similar to primary psychopathy) and antisocial (similar to secondary psychopathy) SDIs. No significant differences were found between the psychopathic and antisocial group on the TAS-20 subscales and total score, suggesting that alexithymia was related to both the interpersonal/affective and impulsive/antisocial features of psychopathy. The study conducted by Gori et al. (2014) has several limitations, which were addressed in the current study. First, they used a self-report measure of psychopathy, which has known disadvantages for use with psychopaths due to its susceptibility to dishonesty, socially desirable response bias and lack of self-reflection. In contrast, we used the Psychopathy Checklist, considered the ‘gold standard’ in the assessment of psychopathy, as it is based on extensive semi-structured interview with the participant, rather than self-report. Second, the sample of Gori et al. (2014) study consisted of a heterogeneous group of individuals dependent on different types of drugs (heroin, cocaine, alcohol, cannabis, etc.) and lacked an appropriate control group for comparisons. In contrast, our study includes a unique group of ‘pure’ (mono-dependent) HDIs, which increases the sample homogeneity and allows more refined between-group comparisons with the control group.

Our findings reveal that in HDIs, alexithymia is uniquely related to the impulsive/antisocial features of psychopathy (PCL:SV Factor 2) but not to the interpersonal/affective psychopathic traits (PCL:SV Factor 1). It is widely acknowledged that difficulties in identifying and processing emotions are related to externalizing traits and behaviors such as impulsivity, aggression, poor behavioral controls and antisocial behaviors, which are among the core characteristics of the impulsive/antisocial dimension of psychopathy. Therefore, the observed relationship between alexithymia and PCL:SV Factor 2 was theoretically expected and consistent with data from previous studies (Kroner and Forth, 1995; Louth et al., 1998; Grieve and Mahar, 2010; Lander et al., 2012). There are several potential explanations for the lack of association between alexithymia and PCL:SV Factor 1 after controlling for the effect of PCL:SV Factor 2. First, it is possible that HDIs with high levels of interpersonal/affective features of psychopathy (similar to primary psychopathy) do not experience difficulties in identifying, differentiating, and verbalizing emotional states. This notion is consistent with findings suggesting that primary psychopaths mask their deficient affective experience by using a rich emotional vocabulary (Del Gaizo and Falkenbach, 2008), which suggests that they may lack the cognitive deficit specific to alexithymia in describing and interpreting emotions. This hypothesis is in line with neuroimaging studies on emotional processing in criminal psychopaths, which have found no performance differences between psychopaths and control participants in emotional tasks but revealed qualitatively distinct patterns of neural processing across groups (Kiehl et al., 2001; Glenn et al., 2009; Contreras-Rodríguez et al., 2013). Overall, these studies reveal decreased activation in limbic areas, combined with increased neocortical activation in criminal psychopaths, suggesting that they may use alternative, compensatory cognitive strategies when processing emotional stimuli. In addition, Contreras-Rodríguez et al. (2013) observed that these compensatory pathways are uniquely related to the interpersonal and affective characteristics of psychopathy (PCL-R Factor 1). Thus, it is possible that individuals high on PCL Factor 1 scores rely more on cognitive strategies when performing emotional tasks in the absence of appropriate input from emotion-related brain areas. In line with these findings, it is possible that individuals with high levels of interpersonal/affective features of psychopathy may verbalize and simulate a wide range of emotions in order to manipulate and exploit others, without actually experiencing these emotions. On the other hand, some of the core personality traits specific to primary psychopathy such as grandiosity and superficiality could compromise their ability to admit certain difficulties in identifying emotions (Kroner and Forth, 1995). Moreover, their inflated sense of self-worth could limit their capacity for self-reflection and thereby prevent them from recognizing and reporting specific impairments in emotion processing. In line with recent studies (Garofalo et al., 2018), the interpersonal/affective traits of psychopathy could be related to diminished capacity for identifying emotions, but this is not well-captured by the TAS-20, which has been criticized for its exclusive reliance on the individual’s capacity for self-reflection (Lane et al., 2015).

In summary, our data reveal that HDIs with higher levels of psychopathy face more difficulties in identifying and verbalizing emotions, suggesting that alexithymia could be considered an underlying risk factor for both psychopathy and heroin dependence. The identification of potential mechanisms linking psychopathy and SUDs has important clinical implications for prevention and treatment of SUDs and reduction of criminal behaviors in SDIs. Although, there is no evidence-based treatment specifically targeting alexithymia, researchers have emphasized the growing need of developing treatment interventions targeting this trait (Vanheule et al., 2011; Samur et al., 2013). Our findings suggest that alexithymia could play a key role in the concurrent development of SUDs and psychopathy. Thus, the implementation of treatment interventions targeting the identification and regulation of emotions could be of particular importance when working with SDIs with comorbid psychopathy, who are often considered “treatment-resistant.” Such interventions could prove to be successful in reducing relapse rates in SDIs, which are consistently predicted by higher levels of negative affect (Baker et al., 2004; Kadam et al., 2017). Nevertheless, our findings require further investigation in larger samples of HDIs. In addition, future studies should examine whether these relationships are observed in other types of SUDs (e.g., alcohol-, cannabis-, stimulant use disorders) and in different stages of the addiction cycle or whether they are uniquely associated with opioid use disorder and the protracted abstinence stage of addiction.

Finally, a few important limitations need to be considered. A key limitation of this study is the small sample size, which impedes the generalizability of our findings and prevents us from evaluating the potentially mediating effect of alexithymia on the relationship between psychopathy and SUDs. In addition, our sample size was insufficient to study possible sex differences in the pattern of relationships between alexithymia and psychopathy. In line with our recent study, suggesting that sex differences in psychopathy predict different types of aggression (Thomson et al., 2019), it would be useful to examine if sex moderates the associations of psychopathy, alexithymia, and substance use. Our research was a first step in studying the relationship between alexithymia and psychopathy in a relatively homogenous group of heroin mono-dependent individuals and additional studies with more participants are needed. Another limitation is related to the use of the TAS-20 to measure alexithymia. Given that a reduced capacity for self-reflection is considered one of the core features of alexithymia, measuring it with self-report questionnaires is problematic as it relies exclusively on the individual’s capacity to reflect on his/hers inner emotional states and difficulties and on the veracity of self-report (Lane et al., 2015). Although TAS-20 continues to be regarded as the most reliable measure of alexithymia, a multi-method measurement has been recommended, which combines clinical interviews with self-report scales such as the TAS-20 in order to increase the validity of the results (Loas et al., 2017). Future research could use clinical interviews for assessing emotional difficulties such as the Toronto Structured Interview for Alexithymia (Bagby et al., 2006), in conjunction with neurocognitive tasks of emotional processing in order to obtain a valid and objective measure of the quality of emotional processing. Lastly, the current findings are specific to ‘pure’ opioid addiction and could not be generalized to individuals dependent on other types of drugs, including opiate users who have concurrent dependence on other classes of drugs. Further studies should examine these relationships with other drugs of abuse and evaluate the role of alexithymia in different types of SUDs comorbid with psychopathy in order to identify potential substance-specific differences.

## Conclusion

Our findings underscore the importance of emotional deficits in heroin dependence and suggest that alexithymia could be considered a potential common mechanism underlying both psychopathy and SUDs. Assessing and addressing difficulties in emotional processing could have significant implications for relapse prevention and intervention programs for SUDs as well as in interventions aimed to decrease criminal behaviors among SDIs.

## Ethics Statement

All subjects gave written informed consent in accordance with the Declaration of Helsinki. The protocol was approved by the Institutional Review Boards of Virginia Commonwealth University and the Medical University in Sofia on behalf of the Bulgarian Addictions Institute.

## Author Contributions

EP, NA, and JV conceived the study. EP performed the statistical analyses and drafted the analysis, Results and Discussion sections, and collected and managed the data with the assistance of the Bulgarian Addiction Institute’s research team. EP and SS drafted the introduction section. JV supervised the data collection and analyses and drafted portions of the manuscript. All authors discussed the results and contributed to the final manuscript.

## Conflict of Interest

The authors declare that the research was conducted in the absence of any commercial or financial relationships that could be construed as a potential conflict of interest.
